# Evaluating the relationship between citation set size, team size and screening methods used in systematic reviews: a cross-sectional study

**DOI:** 10.1186/s12874-021-01335-5

**Published:** 2021-07-08

**Authors:** Katie O’Hearn, Cameron MacDonald, Anne Tsampalieros, Leo Kadota, Ryan Sandarage, Supun Kotteduwa Jayawarden, Michele Datko, John M. Reynolds, Thanh Bui, Shagufta Sultan, Margaret Sampson, Misty Pratt, Nick Barrowman, Nassr Nama, Matthew Page, James Dayre McNally

**Affiliations:** 1 CHEO Research Institute, Ottawa, ON Canada; 2 School of Engineering and Applied Sciences, McMaster University, Hamilton, ON Canada; 3 Department of Pediatrics, Faculty of Medicine, University of Ottawa, Ottawa, ON Canada; 4 Faculty of Medicine, University of British Columbia, Vancouver, BC Canada; 5 ECRI Information Center, ECRI, Plymouth Meeting, PA USA; 6 Calder Memorial Library, University of Miami Miller School of Medicine, MLIS, Miami, FL USA; 7 Faculty of Arts & Science, University of Toronto, Toronto, ON Canada; 8 Therapeutic Products Directorate, Health Canada, Ottawa, ON Canada; 9 Library Services, CHEO, Ottawa, ON Canada; 10 Department of Pediatrics, Faculty of Medicine, University of British Columbia, Vancouver, BC Canada; 11 School of Public Health and Preventive Medicine, Monash University, Melbourne, Australia; 12 Department of Pediatrics, CHEO, 401 Smyth Road, ON K1H 8L1 Ottawa, Canada

**Keywords:** Systematic reviews, Scoping reviews, Crowdsourcing, Machine learning

## Abstract

**Background:**

Standard practice for conducting systematic reviews (SRs) is time consuming and involves the study team screening hundreds or thousands of citations. As the volume of medical literature grows, the citation set sizes and corresponding screening efforts increase. While larger team size and alternate screening methods have the potential to reduce workload and decrease SR completion times, it is unknown whether investigators adapt team size or methods in response to citation set sizes. Using a cross-sectional design, we sought to understand how citation set size impacts (1) the total number of authors or individuals contributing to screening and (2) screening methods.

**Methods:**

MEDLINE was searched in April 2019 for SRs on any health topic. A total of 1880 unique publications were identified and sorted into five citation set size categories (after deduplication): < 1,000, 1,001–2,500, 2,501–5,000, 5,001–10,000, and > 10,000. A random sample of 259 SRs were selected (~ 50 per category) for data extraction and analysis.

**Results:**

With the exception of the pairwise t test comparing the under 1000 and over 10,000 categories (median 5 vs. 6, *p* = 0.049) no statistically significant relationship was evident between author number and citation set size. While visual inspection was suggestive, statistical testing did not consistently identify a relationship between citation set size and number of screeners (title-abstract, full text) or data extractors. However, logistic regression identified investigators were significantly more likely to deviate from gold-standard screening methods (i.e. independent duplicate screening) with larger citation sets. For every doubling of citation size, the odds of using gold-standard screening decreased by 15 and 20% at title-abstract and full text review, respectively. Finally, few SRs reported using crowdsourcing (*n* = 2) or computer-assisted screening (*n* = 1).

**Conclusions:**

Large citation set sizes present a challenge to SR teams, especially when faced with time-sensitive health policy questions. Our study suggests that with increasing citation set size, authors are less likely to adhere to gold-standard screening methods. It is possible that adjunct screening methods, such as crowdsourcing (large team) and computer-assisted technologies, may provide a viable solution for authors to complete their SRs in a timely manner.

**Supplementary Information:**

The online version contains supplementary material available at 10.1186/s12874-021-01335-5.

## Introduction

Systematic reviews (SRs) are often placed at the top of the evidence pyramid due to their systematic methods and consideration of the entire body of evidence on a topic [1]. Unfortunately, the standard practice of relying on small teams of individuals to perform time-consuming tasks, such as screening thousands of abstracts, retrieving and reviewing hundreds of full-text articles, and data extraction, often leads to considerable delays between study initiation and completion [2]. This issue is further compounded by the recent exponential growth in scientific literature [3], resulting in a larger number of citations at each stage of the SR process and a higher workload for each team member. Consequently, SRs are not initiated due to concerns about feasibility, abandoned along the way, take years to finish, or are out of date shortly following publication [4].

There is significant interest in methods that can increase the speed and efficiency of SR completion [5–14]. Approaches intended to increase efficiency include computer-assisted screening (natural language processing or machine learning) [10–13], screening by a single reviewer [9], or screening of the title without the abstract [8]. While these methods can reduce the workload per reviewer or decrease the time to SR completion, there are concerns these methodological approaches may compromise SR quality [9, 15–17]. For example, some authors have found a 7%-10% loss in sensitivity when single reviewer screening is used [9].

One potential approach to accelerate the completion of large SRs, while adhering to the gold standard methodology of two independent assessments per citation, would be to involve a larger team. This would reduce the workload per reviewer, facilitating faster and potentially more valid and reliable work [18, 19]. For example, our team of almost twenty reviewers completed four systematic reviews on mask decontamination during the COVID-19 pandemic, in an average time of two weeks from protocol development to manuscript dissemination on the Open Science Framework [20–23]. This large team or crowdsourcing approach can be helpful when SRs involve many tasks (e.g. large citation set sizes) or when investigators need to complete an SR at a much quicker pace.

Despite these promising methods, the extent to which investigators adapt team sizes or methods for a SR in response to larger citation set sizes is unknown. Since 1950, the number of authors on MEDLINE-indexed papers has tripled in response to the increasing complexity of research [24], which could suggest that SR teams are growing in relation to citation workload. The aim of this study was to understand how citation set size impacts the approach to conducting SRs, specifically the impact on authorship, team size and systematic review screening methods. Our primary objective was to evaluate the extent to which SR screening workload (initial citation set size) influences the team size. The secondary objective was to report on the influence of citation set size on the application of crowdsourcing, machine learning and non-standard (i.e. single assessment) screening methods.

## Methods

This was a cross-sectional study of SRs in the area of health/medicine indexed in MEDLINE in April 2019. The study protocol is available at https://osf.io/w57ts/) and adheres closely to the standard methodology for systematic reviews.

### Eligibility criteria

Systematic and scoping reviews indexed in MEDLINE in April 2019 were included in this study if they met the PRISMA-P definition of a SR [25]: they demonstrated a knowledge synthesis effort, their stated objective was to summarise evidence from multiple studies, explicit methodologies were described, and the report represented an original study (i.e. not a protocol or a summary of published findings). Scoping reviews and Health Technology Assessments (HTAs) met this definition [26]. We included SRs on any health/medicine-related topic (i.e. biomedical, technological, social) that included any type of studies (i.e. qualitative and quantitative). We excluded narrative reviews, non-systematic literature reviews, rapid reviews, and umbrella reviews. We excluded publications that reported two different studies, if the main objective was not the SR (e.g. an SR was used to inform the main objective of the study). We also excluded SRs written in a language other than English. The inclusion and exclusion criteria are shown in Supplementary Material 1.

### Search

We searched for SRs indexed in MEDLINE throughout one month (April 2019). We searched Ovid MEDLINE In-Process & Other Non-Indexed Citations and Ovid MEDLINE 1946 to May 14, 2019, using the search strategy reported by Moher et al. [27]: 1. 201,904$.ed.; 2. limit 1 to English; 3. 2 and (cochrane database of systematic reviews.jn. or search.tw. or metaanalysis.pt. or medline.tw. or systematic review.tw. or ((metaanalysis.mp,pt. or review.pt. or search$.tw.) and methods.ab.)). From this search, we selected those articles indexed in April 2019. Records were downloaded and imported into Reference Manager.

### Study selection

Citation screening was performed using insightScope (www.insightscope.ca), a web-based platform that allows the creation of a large, online team to facilitate rapid citation screening [22, 28]. After piloting screening on an initial random selection of 400 citations, we sought to recruit ≥ 10 additional team members. Before citation screening was initiated the reviewers read the study protocol and were required to achieve sensitivity above 80% on a test set of 50 randomly selected citations (40% true positives) [28]. Ten individuals completed and passed the test set, with an average sensitivity and specificity of 94 and 69%, respectively. Kappa values were calculated for title-abstract and full text screening using the Fleiss approach; values between 0.6 and 0.8 are considered moderate, and values > 0.8 are considered strong [29].

Citations at both title/abstract and full-text levels were assessed by two reviewers independently, with conflict resolution performed by one of the three study leads. For all SRs meeting our inclusion criteria we recorded initial citation set size, defined as the total number of citations screened at the first level of the SR, after duplicate removal. SRs that did not report removing duplicates were included only if the number of citations screened at the first screening level was clearly reported. The included SRs were then sorted into 5 citation set size categories: < 1,000, 1,001–2,500, 2,501–5,000, 5,001–10,000, and > 10,000. These categories were chosen based on results from Page et al. [30], and intended to approximately represent the 50^th^, 75^th^, 90^th^ and 95^th^ percentile cut-offs.

A random sample of 50 citations from each category was selected for further data extraction (no formal sample size calculation was performed). The citation list for each category was imported into Microsoft Excel and each study was assigned a random number using the Generate Random Number feature; citations were sorted by the random number and the first 50 were selected for data extraction. SRs that did not clearly report the size of the initial citation set screened (i.e. the number of citations screened at the first level of screening, post-duplicate removal) were excluded from further analysis.

### Data extraction and verification

A data extraction tool was developed by the study leads using KNACK software (knack.com) and piloted on 10 randomly selected eligible SRs (See Supplementary Material 2 for data extraction variables) The three study leads and seven reviewers contributed to data extraction. Prior to initiating data extraction, reviewers attended a one-hour interactive training webinar. The 250 SRs were then divided among the study team and extracted independently in duplicate. After extraction was completed for a given SR, both team members reviewed conflicting answers and corrected any errors in the data they had entered. Once the data extracted from each SR had been verified by both original extractors one of the study leads resolved outstanding conflicts.

Outcome data is reported for the 50 random SRs from the five initial citation set size categories. The primary objective was to determine the relationship between citation set size and team size, which was evaluated using both author number and number of screeners (when stated). The number of authors was determined based on the authors named on the paper, and individuals listed as part of group authorship (if applicable). The number of screeners was defined as the number of individuals reported in the manuscript or acknowledgments to have contributed to citation screening (for a given screening level). The average workload per screener for a given screening level was calculated using only those SRs that reported using two assessments per citation, regardless of independence. Gold-standard methodology was defined as two (or more) independent assessments per citation.

### Statistical analysis

Characteristics of the SRs were summarized using descriptive statistics. Continuous variables were described using mean and standard deviation (SD) if normally distributed, or median and interquartile range (IQR) for non-normal distributions. Categorical variables were described using frequencies and percentages. Pearson correlation was used to quantify a relationship between citation set size and listed author number. Pairwise t-tests, corrected for multiple comparisons using a Holm correction, were used to compare listed author numbers by citation set size category. Logistic regression was used for the following outcomes: screener number (> 2 screeners vs 1 or 2 screeners) as the dependent variable, gold standard methodology at the title/abstract level as the dependent variable, as well as gold standard methodology at the full-text level as the dependent variable. A Chi-squared test was performed to test for differences in proportions. The independent variable, citation set size, was log transformed as it varied by several orders of magnitude. As categorization of citations by abstract citation set size was potentially less applicable for the evaluation of relationships at the full-text level we also evaluated for relationships by grouping citations based on terciles. All analyses were performed using R statistical software version 3.6.2 [31].

### Changes from the original protocol

There were three major changes from the original study protocol. First, the original protocol included an objective proposing to determine whether there was a relationship between citation set size and time required for SR completion. Due to a combination of significant missing data, and concerns about misclassification, this objective was not pursued. Second, a decision was made to evaluate differences in study screening and extraction approaches using not only the 5 citation size categories assigned based on the initial citation set sizes, but terciles corresponding to the citation set sizes at full text review and data extraction. Finally, given the small number of SRs that used non-standard screening methods (e.g. crowdsourcing or computer assisted screening) no further analysis was possible.

## Results

### Search results

**Fig. 1:**
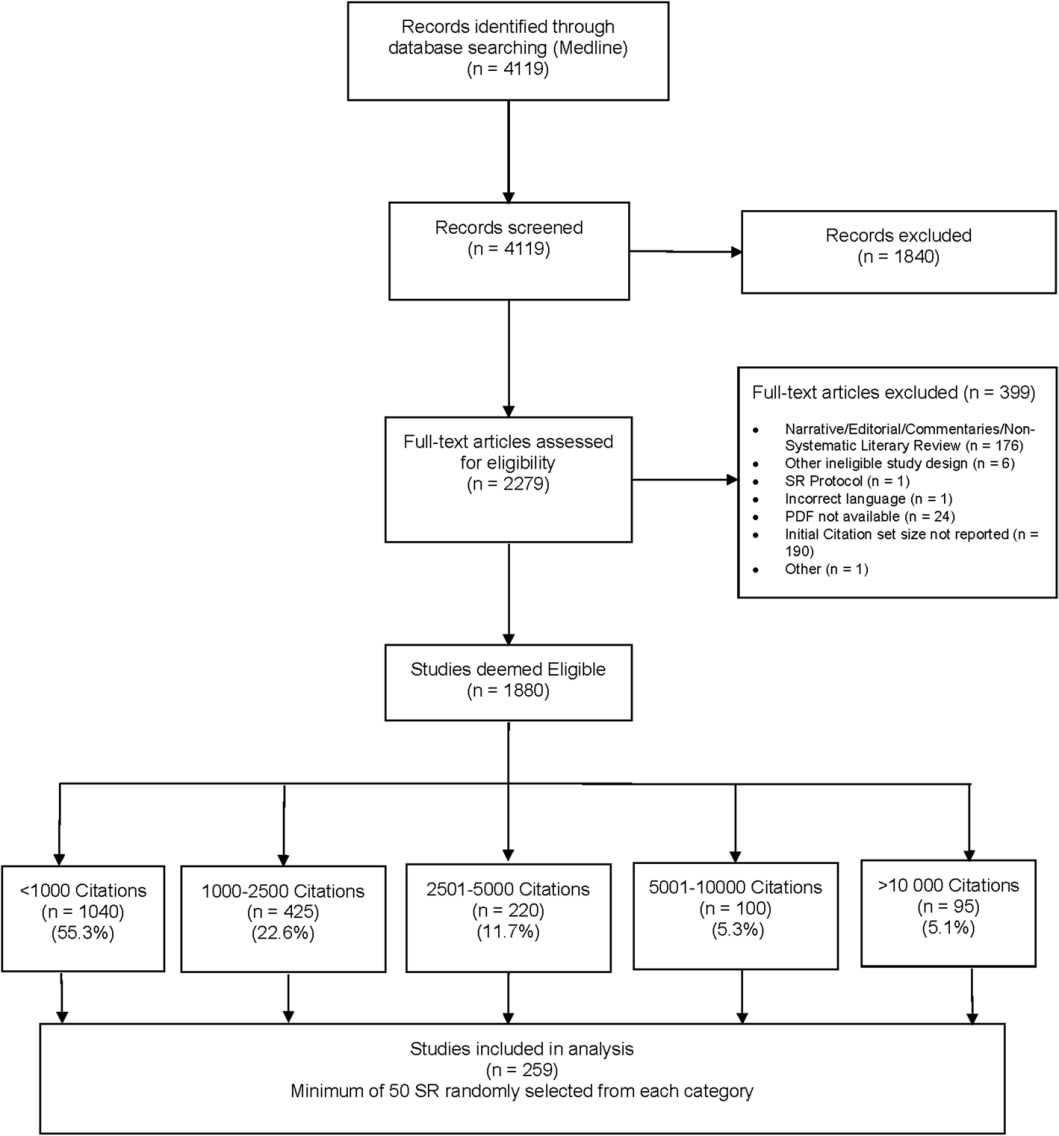
PRISMA Diagram

A total of 4,119 citations were retrieved from the MEDLINE search. Title and abstract screening excluded 1,840, with the review team achieving a kappa of 0.81. Review of the remaining 2,279 citations at full text identified 1,880 eligible SRs, with an overall kappa of 0.67. An overview of the screening process, results and reasons for exclusions are shown in the PRISMA diagram (Fig. 1). Upon more detailed review during data extraction, nine of the 250 randomly selected SRs were reclassified into different citation set size categories. To achieve a minimum of 50 SRs in each size category, an additional nine citations were randomly selected, providing a final sample size of 259 SRs.

### Epidemiology of the included systematic reviews

Table 1 provides a summary of the characteristics of the 259 systematic reviews (see Supplementary Material 3 for a summary of the number of SRs included in each subsection of the results). The overall median initial screening set size for the 259 SRs was 3,585 (IQR: 1,350 to 6,700) with a maximum of 215,744. The median (IQR) number of citations for each initial citation set size category is presented in Table 2. For the full cohort of 259 articles, the median author number was 6 (IQR: 4 to 8), with a maximum of 26. Three levels of screening (title, abstract, full text) were performed in 42 (16.2%) of the SRs. Authors of all 259 SRs completed full-text screening, and all but one completed data extraction (no eligible studies were identified so no data collection occurred). Authors of two SRs used crowdsourcing during title/abstract screening [32, 33], and authors of one SR used computer-assisted screening during title only screening [34].

**Table 1:** Epidemiology of the included systematic reviews

Overall cohort (*N* = 259)	
**Publication year, N (%)**	
*2017* *2018* *2019* Missing/other	8 (3.1) 1443 (55.62) 107 (41.3) 1 (0,3)
**Country of origin, N (%)**	
*United States* *United Kingdom* *China* *Australia* *Canada* *The Netherlands* *Germany* *Other*	43 (16.6) 42 (16.2) 29 (11.2) 23 (8.9) 21 (8.1) 9 (3.5) 6 (2.3) 86 (33.2)
**Review type, N (%)**	
*Systematic* *Scoping* *Integrative* *Health technology assessment* Missing/other	247 (95.4) 7 (2.7) 3 (1.2) 2 (0.8) 2 (0.8)
**Population, N (%)**	
*Adult* *Pediatric* *Both/Unclear* *Animal*	131 (50.6) 29 (11.2) 96 (37.0) 3 (1.2)
**Focus of systematic review, N (%)**^**a**^	
*Therapeutic* *Epidemiology* *Diagnosis* *Other*^*a*^	120 (46.3) 82 (31.7) 201 (7.70.8) 37 (14.32)
**Cochrane review, N (%)**	
*Yes*	31 (12.0)
*No*	228 (88.0)
**Update, N (%)**	
*Yes*	28 (10.8)
*No*	231 (89.2)
**Registered protocol, N (%)**	
*Yes*	94 (36.3)
*No*	165 (63.7)
**Alternate screening methodologies used, N (%)**	
*Crowdsourcing*	2 (0.7)
*Computer assisted screening*	1 (0.3)

^a^Examples of other include education, review of psychometric properties, barriers analysis, cost of illness

**Table 2:** Overall citation set size

Overall (*n* = 259)		
**Initial citation set size category**	**n (%)**	**Median (IQR) citation set size**
< 1000	50 (19.3)	284 (166, 572)
1000–2500	50 (19.3)	1663 (1318, 2007)
2500–5000	58 (22.4)	3574 (3094, 3999)
5000–10,000	50 (19.3)	5832 (5543, 6731)
> 10,000	51 (19.7)	15,152 (12,247, 23,192)

### Relationship between number of authors and citation set size

**Fig. 2:**
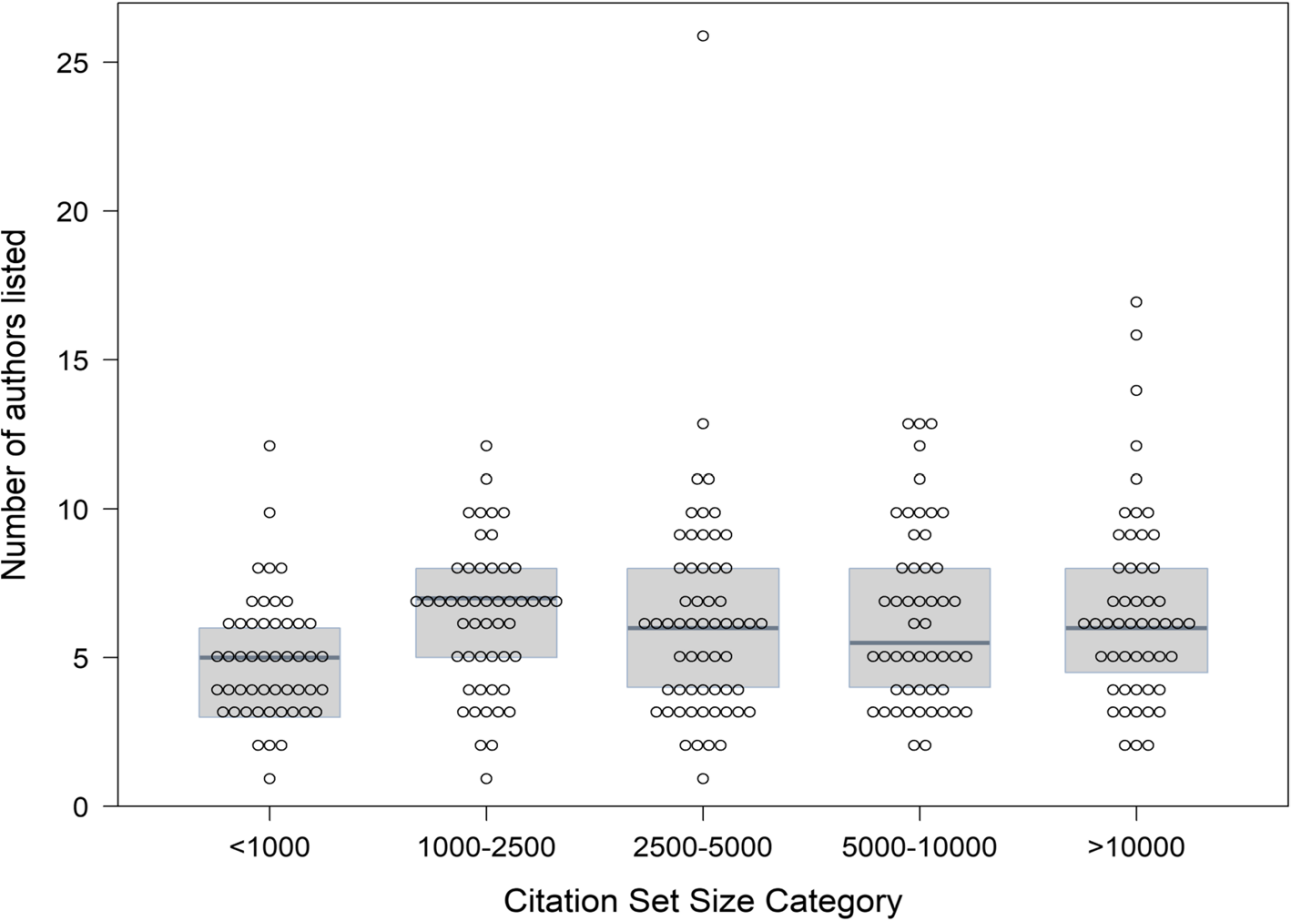
Listed author number by initial citation set size category (*N* = 259). The median (IQR) number of authors listed by citation set size category was as follows: < 1,000: 5.0 (3.2, 6.0); 1,000–2,500: 7.0 (5.0, 8.0): 2,500–5,000: 6.0 (4.0, 8.0): 5,000–10,000: 5.5 (4.0, 8.0) and > 10,000: 6.0 (4.5, 8.0). In pairwise t tests, corrected for multiple comparisons using Holm’s method, only three comparisons yielded a p-value less than 0.2: < 1000 vs 1,000–2,500 (*p* value = 0.18); < 1000 vs 5,000–10,000 (*p* value = 0.15); and < 1000 vs > 10,000 (*p* value = 0.046)

The median author number and IQR for each initial citation set size is presented in Fig. 2. Confirming visual inspection, application of pairwise t test with Holm’s correction, identified that the only between-group comparison with a p-value under 0.05 was between the < 1000 (median 5, IQR: 3.2, 6.0) vs > 10,000 citation set size categories (median 6, IQR: 4.5, 8.0); *p* = 0.049). Further, when examined through Pearson correlation using citation set size as a continuous value, there was little evidence for a relationship between initial citation set size and number of authors (*r* = 0.03, (95% CI: -0.09, 0.15, *p* = 0.63); this finding was unchanged after removing three outliers (*r* = 0.02, 95% CI: -0.13, 0.17, *p* = 0.78).

### Relationship between number of screeners and citation set size

**Fig. 3:**
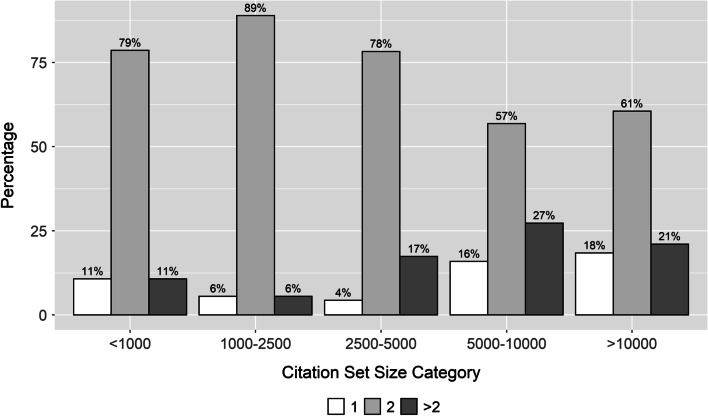
Total number of screeners who contributed to title/abstract screening by initial citation set size category (*N* = 192). The above figure presents the percentage of SRs with teams of 1 (white), 2 (light grey) or > 2 (dark grey) screeners by initial citation set size category

We identified 192 SRs (75%) reporting the total number of screeners contributing to the initial level of screening. The median number of screeners was 2 (IQR: 2 to 2) with the full range from 1 to 24. In total, 21 (11%) SRs reported a single screener performing all of the initial level of screening; 138 (72%) reported a two-screener team, and 33 (17%) reported utilizing a team of more than two screeners. Figure 3 presents the percentage of SRs with teams of 1, 2 or > 2 screeners by initial citation set size category. There appeared to be a greater percentage of SRs that used > 2 screeners in larger citation size categories. The relationship between log initial citation set size and screener number > 2 is illustrated in Supplementary Material 4. In a logistic regression model, the odds ratio between log_2_ citation set size and screener number > 2 was 1.16 (95% CI 0.95, 1.44), *p* = 0.15. An odds ratio of this magnitude would imply that for every doubling in citation set size the odds of using more than two screeners would increase by 16%. When SR initial citation set size was dichotomized into categories representing above or below 2,500, the proportion of SR’s with > 2,500 citations and using > 2 screeners was 21.9% (28/128) compared to 7.8% (5/64) in the smaller citation set size group, *p* = 0.03.

### Average workload per screener

There were 156 SRs that reported using two assessments per citation during title/abstract screening and provided a citation set size at the title/abstract level. As shown in Table 3, there is a strong relationship between workload per screener at the title/abstract level and initial citation set size category. Full-text workload per screener by initial citation set size is presented in Table 4. Again, the workload per screener was higher in the larger citation set size categories. For example, the large citation set size category required 117 (53, 345) citations per screener, with a median of only 40 (20, 58) in the smallest initial citation set size category.

**Table 3:** Workload per screener at title/abstract by initial citation set size category

Overall (*n* = 156)			
**Initial citation set size category**	**n (%)**	**Median (IQR) citation number**	**Median (IQR) number of citations reviewed per screener during title/abstract screening**^a,b^
< 1000	24 (15.4)	274 (175, 571)	265 (140, 445)132 (70, 222)
1000–2500	33 (21.2)	1597 (1307, 2082)	1597 (1161, 2082)798 (580, 1041)
2500–5000	36 (23.1)	3362 (2960, 3971)	3222 (2332, 3880)1611 (1166, 1940)
5000–10,000	34 (21.8)	5832 (5263, 6858)	5328 (2803, 6660)2664 (1402, 3330)
> 10,000	29 (18.6)	13,399 (2086, 23,847)	13,033 (2086, 18,311)6516 (1043, 9156)

^a^For most reviews, two screeners were assigned. When there were more than two screeners, not all screeners reviewed each citation

^b^Possible to have more than 1 screener per citation

**Table 4:** Full text workload per screener by initial citation set size category

Overall (*n* = 139)			
**Initial citation set size category**	**n (%)**	**Median (IQR) number of records for full text screening**	**Median (IQR) number of full text records reviewed per screener during full text screening**^**a**^
< 1000	22 (15.8)	40 (28, 58)	40 (20, 58)20 (10, 29)
1000–2500	27 (19.4)	82 (44, 178)	79 (44, 178)40 (22, 89)
2500–5000	34 (24.5)	98 (30, 222)	74 (28, 163)37 (14, 81)
5000–10,000	28 (20.1)	118 (51, 194)	107 (36, 158)53 (18, 79)
> 10,000	28 (20.1)	131 (72, 640)	117 (53, 345)58 (26, 173)

^a^For most reviews, two screeners were assigned. When there were more than two screeners, not all screeners reviewed each citation

### Methodological approach to title and title/abstract screening

There were 116 (44.8%) SRs that adhered to the gold-standard methodology for both citation screening levels performed (i.e. two or more independent assessments per citation).

Title only screening was performed in 42 (16.2%) of the 259 SRs. Of these, 45.2% (*N* = 19/42) reported using gold-standard methodology for title screening; 4.8% (*N* = 2/42) reported using two assessments per citation but independence was not reported or was unclear; 19.0% (*N* = 8/42) of SRs reported using a single assessment per citation; and 2.4% (*N* = 1/42) of the SRs reported using computer-assisted screening. Finally, for the remaining 28.6 (*N* = 12/42), the screening method used was not reported or was unclear.

During title/abstract screening, 163 (62.9%) SRs utilized gold-standard methodology. The screening approach used at the title/abstract level by initial citation set size category is presented in Table 5. There appeared to be a smaller proportion of SRs in the larger citation set size categories reporting standard methodology. In a logistic regression model examining the association between log_2_ initial citation set size and gold-standard approach to screening, the odds ratio was 0.85 (95% CI 0.71, 1.00), *p* = 0.05 (see Supplementary Material 5). An odds ratio of this magnitude implies that for every doubling of the initial citation set size, there is a 15% decrease in the odds of using the gold standard approach. When dichotomizing SRs into large and small size categories, the proportion using gold-standard methodology was 98/136 (72.1%), when the initial citation set was > 2,500 compared to 65/78 (83.3%) when the initial citation set size was ≤ 2,500 (*p* = 0.09).

**Table 5:** Distribution of methodology used during title/abstract screening by initial citation set size category (*n* = 214)^a^

Citation set size category	N	Gold-standard methodology	Two assessments/citation, not independent or not stated	Single assessment/citation
		***N* = 163**	***N* = 25**	***N* = 26**
		**N (%)**	**N (%)**	**N (%)**
< 1000	34	28 (82.4)	4 (11.8)	2 (5.9)
1000–2500	44	37 (84.1)	5 (11.4)	2 (4.5)
2500–5000	49	35 (71.4)	7 (14.3)	7 (14.2)
5000–10,000	46	34 (73.9)	2 (4.3)	10 (21.7)
> 10,000	41	29 (70.7)	7 (17.1)	5 (12.1)
N	214^a^	163	25	26

^a^This table is limited to the included studies that reported their screening approach thus the total number of SRs included is 214

### Methodological approach to full-text screening

For full-text screening, the methodology used is presented in Table 6. Terciles were created for the 186 SRs based on the number of citations retained for full-text screening. When evaluating the relationship between screening methodology and full-text set size, there was a higher percentage of SRs using two (or more) reviewers in the smaller citation set size tercile (see Supplementary Material 6). In a logistic regression model examining the association between log_2_ full-text set size and gold-standard approach to screening, the odds ratio was 0.80 (95% CI 0.65, 0.99) implying for every doubling of the set size at the full-text level there was a 20% decrease in the odds of using the gold standard approach (see Supplementary Material 7).

**Table 6:** Distribution of methodology used during full text screening by initial citation set size category (*n* = 186)^a^

Citation set size category	N	Full text screening approach (Other)	Single reviewer	Two (or more) reviewers, independence stated	Two (or more) reviewers, not independent or not stated
		***N* = 3**	***N* = 14**	***N* = 147**	***N* = 22**
		**N (%)**	**N (%)**	**N (%)**	**N (%)**
** < 1000**	29	0 (0.0)	1 (3.4)	23 (79.3)	5 (17.2)
**1000–2500**	40	1 (2.5)	3 (7.5)	33 (82.5)	3 (7.5)
**2500–5000**	42	0 (0.0)	2 (4.8)	36 (85.7)	4 (9.5)
**5000–10,000**	35	1 (2.9)	5 (14.3)	27 (77.1)	2 (5.7)
** > 10,000**	40	1 (2.5)	3 (7.5)	28 (70.0)	8 (20.0)
**N**	1860	3	14	147	22

^a^This table is limited to the included studies that reported their screening approach thus the total number of SRs included is 186

### Methodological approach to data extraction

A total of 141 (54.4%) of the 259 SRs reported that the majority of all data was extracted in duplicate. Supplementary Material 8 summarizes the methodology used for data extraction by initial citation set size category. The number of articles in each SR at data extraction was then used to create terciles, with a median (IQR) number of citations 40.0 (range: 25.0, 72.0). No relationship was observed between the number of individuals who contributed to data extraction and initial citation set size, or between the number of individuals who contributed to data extraction and number of articles for data extraction, based on terciles (see Supplementary Material 9).

## Discussion

Using a cross-sectional study design, we sought to identify a large representative sample of health-related systematic reviews to evaluate the relationship between citation set size, team size and screening methods. We identified considerable variability in citation set size, suggesting that there are thousands to potentially tens of thousands of SRs published each year with initial citation set sizes exceeding 5,000. Despite this wide-ranging variability in initial citation set sizes, we did not observe a discernable relationship between the number of authors or the average number of screeners and citation set size. Further, few studies appeared to use or report adjunct screening methods including crowdsourcing or computer-assisted screening. Finally, this work provides evidence that SR teams are more likely to deviate from accepted gold standard screening approaches when faced with larger citations sizes.

Our search of a single health database identified approximately 2,000 systematic reviews published in a one-month timeframe. This finding suggests that at least ~ 25,000 SRs are published each year, which is likely a considerable underestimate if one considers other publication databases, unpublished work, and reviews from other disciplines within the natural and applied sciences. In addition, through comparison with findings from similar studies, this work suggests that the rate of SR publication is rapidly increasing. For example, Page et al*.*, [30] using very similar methodology and SR definitions, identified 682 systematic reviews indexed in MEDLINE during a single month in 2014; these numbers would suggest an approximate three-fold increase in a five-year period. This is further supported by Alabousi et al. who reported that publication rates for diagnostic imaging-related SRs have increased more than ten-fold between 2000 and 2016 [35].

In addition to reporting on the current rate of SR publication this work also provided valuable information on the distribution of citation set sizes. During full-text screening, the SRs were placed into five categories, based on the post de-duplication citation set size. While just over half of the studies have citation set sizes at or below 1,000, we also found that a fair proportion (10%) had large citation set sizes (> 5,000). Given the number of SRs performed each year there are many thousands of SRs performed annually with citation set sizes in excess of 5,000 or 10,000. As the growth rate of published scientific literature increases (recent estimates have found a growth rate anywhere from 3.5 to 9% per year [36, 37]) it is likely that initial citation sets sizes could continue to grow unless countered by improvements in citation coding and search methodology. Investigative teams will need to find means for accommodating large citation sets, in an era when timely medical and healthcare policy decisions are needed. Strategies to accommodate large SRs may be to streamline the topic and research questions or focus search strategies using validated filters to limit certain study types or topics [38].

A logical response to increased workload is expansion of team size, and we sought to explore the relationship between citation set size and authorship. Studies are now being authored by dozens, hundreds and sometimes even more than 1,000 authors, a phenomenon known as “hyperauthorship” [39]. This trend appears to be most apparent in the field of physical sciences, where open data and the need for collaboration has prompted authors to combine resources [39]. The number of authors per publication in the biomedical literature also appears to be growing [40]. The need for a multidisciplinary approach and the desire to collaborate to increase the chances for publication may be the driving factor in longer author lists [41]. Despite these observations from the scientific literature, this study did not find evidence of a relationship between citation set size and author number. Performing the analysis using the reported number of screeners as opposed to author number again did not suggest a relationship between screening team size and citation set size categories. However, a more detailed inspection demonstrated that SRs with larger citation set size were less likely to use two screeners, with an increase in the proportion of SRs using a single screener approach.

In addition to assessing total screeners by citation set size, we also evaluated the relationship between citation set size and the use of adjunctive or non-standard screening approaches. While not statistically significant, our analysis did suggest that for every doubling of citation set size, there was a decrease in the odds of using gold standard methodology; implying that with increasing workload, adherence to best practices declines. Importantly, our ability to evaluate for differences was potentially limited by the observation that more SRs failed to report their methodology (16%) than indicated using single assessment per citation (10%). Single reviewer screening has been shown to miss substantially more studies than conventional double screening [42].

In addition to evaluating whether investigators performing large reviews adapt through team size, we also evaluated the frequency of application of other methodologies intended to reduce workload. Over the past two decades, there has been considerable interest in the ability of natural language processing to assist with abstract screening, and available evidence suggests that with the proper application the human screening burden can be reduced and time saved [43, 44]. Yet, despite a significant number of publications on the topic, and incorporation into a number of common screening platforms, only one of the 259 SRs reported its application. The SR used Cochrane’s RCT Classifier, a machine learning system that can identify RCT (and quasi-RCT) trials and non-RCTs [34]. The surprisingly low uptake of computer-automated screening may relate to a lack of trust in the technology and uncertainty over implementation [45], although our study was not designed to answer this question. Alternatively, SRs may not be reliably reporting the use of automation. Finally, given the sample of SRs used for this analysis had screening performed in 2019 or before, it is possible that a larger proportion of more recently completed or ongoing SRs are utilizing adjunct screening methods.

A second and newer methodological approach intended to reduce the burden on study leads and allow for faster systematic review completion is crowdsourcing. This approach has been validated by our group [7, 46] and others [6, 47–49], demonstrating that large groups of individuals are willing to assist with systematic review tasks. With minimal training, these groups can accurately perform citation screening with sensitivity approaching 100% and work saved in excess of 50%. While crowdsourcing was only identified in two of the 259 SRs (0.8%) it is important to acknowledge that the method has only recently been validated and there are just a few platforms available (Cochrane Crowd, insightScope). Both the identified studies were Cochrane reviews, with one being an update of the original SR [32, 33]. The “crowd” in the original review was made up of students and provided an initial screen of search results to decide whether citations were described as randomized or quasi-randomized. This approach removed 81% of citations retrieved (*N* = 4,742/5,832).

Interestingly, a number of studies have been published suggesting and even validating a hybrid of crowdsourcing and machine learning [50, 51]. As crowdsourcing, with or without machine learning, becomes more mainstream investigators and organizations will have to consider what constitutes a reasonable workload for a crowd member – particularly one seeking to join the team and who might eventually meet criteria for authorship. Our findings on average workload provide a starting point for these investigators, indicating that on average, screeners in a SR assessed approximately 1,000 citations at title/abstract and 50 at full-text. Dividing the work in this fashion would help guarantee screening and extraction occur in a timely manner and may avoid the deterioration in screening accuracy that is anticipated with excessive workloads.

A limitation to this study is that author size for SRs may not accurately represent the number of people contributing to screening and data extraction. It is common for only a subset of authors listed to contribute to screening and data extraction work. While we attempted to account for this through a secondary analysis using the number of stated screeners, misclassification is possible as some study groups may not have described individuals who did not meet authorship criteria. Secondly, we did not assess the potential role of time (i.e. how long it took to screen through each level) as a factor in citation set size and screening methodology, given that most SRs did not report this data. This points to a further limitation in the lack of reporting on SR methodology [52], which could have underestimated the number of SRs using gold-standard screening methods or alternative methods. Further, the small sample size of selected SRs from one month in a single health-related database and uncertainty about the strength of association between alternative screening methodologies and citation set size may have influenced findings. However, our aim was to map SR screening methods within a relevant scope of practice, and these findings are likely an accurate representation of health-based SR literature.

Overall, evidence points to thousands of SRs with large citation sets being published each year. Increasing workload appears to decrease the likelihood that teams will use gold standard approaches to citation screening, which may lead to authors’ missing important studies in their included citation set. To help manage the rising volume of published literature we recommend teams performing large SRs consider increasing their team size to ensure that gold standard methods are adhered to, and results are disseminated in a timely manner.

## Supplementary Information

**Additional file 1: Supplementary Material 1.** Screening Criteria. **Supplemental Material 2.** Data Extraction Variables. **Supplementary Material 3.** Number of systematic reviews included in each section of the reported results. **Supplemental Material 4.** Relationship between initial citation set size (log scale) and using more than 2 screeners for the initial screening level. Dots at 1 (or 0) represent systematic reviews which used (or did not use) more than 2 screeners. The blue curve is a binomial 5-knot restricted cubic spline with a 95% shaded confidence band. The vertical line indicates a citation size of 2500. **Supplementary Material 5.** Relationship between initial citation size (log scale) and using the gold-standard approach for citation screening at the title/abstract level. Dots at 1 (or 0) represent systematic reviews which used (or did not use) the gold-standard approach. The blue curve is a binomial 5-knot restricted cubic spline with a 95% shaded confidence band. The vertical line indicates a citation size of 2500. **Supplementary Material 6.** Distribution of Methodology Used during Full text Screening by Terciles Created Using Full Text Citation Size (*n* = 186)^a^. **Supplementary Material 7.** Relationship between full-text citation size (log scale) and using the gold-standard approach to screening at the full text level. Dots at 1 (or 0) represent systematic reviews which used (or did not use) the gold-standard approach. The blue curve is a binomial 5-knot restricted cubic spline with a 95% shaded confidence band. **Supplementary Material 8.** Methodology Used during Data Extraction by Initial Citation Set Size. **Supplementary Material 9.** Methodology Used during Data Extraction by Terciles Created using Data Extraction Citation Set Size.

## Data Availability

The data that support the findings of this study are available from the corresponding author, upon reasonable request.

## Abbreviations

CHEO: Children’s Hospital of Eastern Ontario
HTA: Health technology assessment
IQR: Interquartile range
PRISMA: Preferred Reporting Items for Systematic review and Meta-Analysis
PRISMA-P: Preferred Reporting Items for Systematic review and Meta-Analysis Protocols
RCT: Randomized controlled trial
SD: Standard deviation
SR: Systematic review
