# Supplementary material for: Evaluating the relationship between citation set size, team size and screening methods used in systematic reviews: a cross-sectional study
Source: BMC Med Res Methodol. 2021 Jul 8;21:142. doi: 10.1186/s12874-021-01335-5 (PMC8264476; doi:10.1186/s12874-021-01335-5)
Supplement: Supplementary file 1 — Additional file 1: Supplementary Material 1. Screening Criteria. Supplemental Material 2. Data Extraction Variables. Supplementary Material 3. Number of systematic reviews included in each section of the reported results. Supplemental Material 4. Relationship between initial citation set size (log scale) and using more than 2 screeners for the initial screening level. Dots at 1 (or 0) represent systematic reviews which used (or did not use) more than 2 screeners. The blue curve is a binomial 5-knot restricted cubic spline with a 95% shaded confidence band. The vertical line indicates a citation size of 2500. Supplementary Material 5. Relationship between initial citation size (log scale) and using the gold-standard approach for citation screening at the title/abstract level. Dots at 1 (or 0) represent systematic reviews which used (or did not use) the gold-standard approach. The blue curve is a binomial 5-knot restricted cubic spline with a 95% shaded confidence band. The vertical line indicates a citation size of 2500. Supplementary Material 6. Distribution of Methodology Used during Full text Screening by Terciles Created Using Full Text Citation Size (n = 186)a. Supplementary Material 7. Relationship between full-text citation size (log scale) and using the gold-standard approach to screening at the full text level. Dots at 1 (or 0) represent systematic reviews which used (or did not use) the gold-standard approach. The blue curve is a binomial 5-knot restricted cubic spline with a 95% shaded confidence band. Supplementary Material 8. Methodology Used during Data Extraction by Initial Citation Set Size. Supplementary Material 9. Methodology Used during Data Extraction by Terciles Created using Data Extraction Citation Set Size. [file 12874_2021_1335_MOESM1_ESM.docx]

**Evaluating the relationship between citation set size, team size and screening methods used in systematic reviews: a cross-sectional study
SUPPLEMENTARY MATERIAL**

**Authors:** Katie O’Hearn^1^, Cameron MacDonald^2^, Anne Tsampalieros^1^, Leo Kadota^3^, Ryan Sandarage^4^, Supun Kotteduwa Jayawarden^4^, Michele Datko^5^, John M. Reynolds^6^, Thanh Bui^7^, Shagufta Sultan^8^, Margaret Sampson^9^, Misty Pratt^1^, Nick Barrowman^1^, Nassr Nama^10^, Matthew Page^11^, James Dayre McNally^1,3^.

^1^Children’s Hospital of Eastern Ontario Research Institute, Ottawa, ON, Canada

^2^School of Engineering and Applied Sciences, McMaster University, Hamilton, ON, Canada

^3^Department of Pediatrics, Faculty of Medicine, University of Ottawa, Ottawa, ON, Canada

^4^Faculty of Medicine, University of British Columbia, Vancouver, BC, Canada

^5^Clinical Evidence Assessment/EPC Information Center, ECRI, PA, USA

^6^Calder Memorial Library, University of Miami Miller School of Medicine, MLIS, Miami, FL, USA

^7^Faculty of Arts & Science, University of Toronto, Toronto, ON, Canada

^8^Therapeutic Products Directorate, Health Canada, Ottawa, ON, Canada

^9^Library Services, Children’s Hospital of Eastern Ontario, Ottawa, ON, Canada

^10^Department of Pediatrics, Faculty of Medicine, University of British Columbia, Vancouver, BC, Canada

^11^School of Public Health and Preventive Medicine, Monash University, Melbourne, Australia

**Corresponding author: Dr. Dayre McNally, Department of Pediatrics, Children’s Hospital of Eastern Ontario** [**dmcnally@cheo.on.ca**](mailto:dmcnally@cheo.on.ca)

**Supplementary Material 1. Screening Criteria**

| **Inclusion Criteria** | **Exclusion Criteria** |
| --- | --- |
| We will include systematic reviews, defined as: (1) explicitly states methods to identify studies (these include search strategies such as search terms used in database search); (2) explicitly states methods of study selection (eligibility criteria/selection process for citation screening), and (3) explicitly states methods of synthesis (or other type of summary of data extraction)  If the SR reports the above criteria, we will retain regardless of method used and/or level of detail reported on search strategy  All questions/topics are eligible  We will include scoping reviews  We will include systematic reviews reported in English | Narrative/non-systematic review (see inclusion criteria for definition of systematic review)  Non-systematic literature reviews with meta-analysis or meta-synthesis  Editorial or letter  "Rapid review" or study or other literature review produced using accelerated / abbreviated SR methods  Overview of reviews (umbrella reviews)  Systematic review with methodological focus which are defined as: a study that included a systematic search, but the focus was to evaluate some aspect of conduct/reporting (e.g. assessments of the extent to which all trials published in a time frame adhered to some guidelines)  Main objective of the study was not the systematic review (e.g. A systematic review was used to inform the main objective of the study)  Protocol for a SR  Language other than English |

**Supplemental Material 2: Data Extraction Variables**

1. Author details from the full MEDLINE record, a count of each:
   1. AU, IN, CN AUID
2. Publication Name
3. Year of Publication (print publication, as reported in manuscript)
4. *Recognition structure:
   - Number of authors (AU) (number)
   - Study group named in author list? (CN)
   - Number of people named in the acknowledgment section who are not listed as AU or IR
5. Corresponding Author Information
   1. Name
   2. Email
   3. Country
6. Type of SR study (categorical)
   1. Systematic Review
   2. Integrative Review
   3. Scoping Review
   4. Health and Tech Assessment
7. Population / Age (categorical)
   1. Pediatric
   2. Adult
   3. Both/Unclear
   4. Animal
8. Number of individuals (authors/acknowledgements) who screened and/or extracted data mentioned in
   1. Methods
   2. Results
   3. Acknowledgements
   4. Other
   5. Not Reported
9. Focus of Study
   1. Therapeutic
   2. Epidemiology
      1. Prevalence/Incidence/Prognosis
      2. Risk Factors/Associations
      3. Other
   3. Diagnosis
   4. Other
10. Eligible study types included in the SR as indicated in the methods
11. RCTs in methods
12. Quasi-RCTs
13. Other controlled experimental studies
14. Observational- Retrospective
15. Observational- Prospective
16. Other
17. Unclear/not stated
18. Ineligible study types included in the SR as indicated in the methods
19. RCTs in methods
20. Quasi-RCTs
21. Other controlled experimental studies
22. Observational- Retrospective
23. Observational- Prospective
24. Other
25. Unclear/not stated
26. Cochrane project (Y/N)
27. Update of a previous review (Y/N)
28. Did authors report they registered a protocol (Y/N)
    1. Site
    2. Number
29. Date of search reported
30. Was the search updated
    1. Was the updated search date reported
31. Date manuscript received reported
32. Date manuscript accepted reported
33. Manuscript published date (as reported by the manuscript)
34. PRISMA or similar flow diagram reported
35. More than one level of screening reported at any stage?
    1. At what stages
       1. Title
       2. Title/Abstract
       3. Full Text
       4. Unclear/Not reported
36. Did the authors explicitly state that the two reviewers ‘independently’ screened records for all stages (Y/N) – if no
    1. Approach to title screening
       1. Two or more reviewers independence stated
       2. Two or more reviewers independence not stated
       3. Single reviewer
       4. Computer assisted screening
       5. Other
       6. Unclear
    2. Approach to title/abstract screening
       1. Two or more reviewers independence stated
       2. Two or more reviewers independence not stated
       3. Single reviewer
       4. Computer assisted screening
       5. Other
       6. Unclear
    3. Approach to Full text screening
       1. Two or more reviewers independence stated
       2. Two or more reviewers independence not stated
       3. Single reviewer
       4. Computer assisted screening
       5. Other
       6. Unclear
37. At what stages was citation set size reported
    1. Initial search results (before duplicate removal)
    2. Title
    3. Title/Abstract
    4. Full Text
    5. Full Text retained
    6. Unclear
38. Measurement of inter-reliability
    1. Kappa for two independent
    2. Text Box for single
39. Total reviewers who contributed to the screening reported (y/n)
    1. Where
40. Mention of Pilot of data extraction tool
41. Data extraction methods
    1. Majority two independent
    2. Majority one author with verification
    3. Majority by only one author
    4. Not reported
    5. Other
42. Was the number of reviewers who performed data extraction reported (Y/N)
    1. How many?
43. At the end how many records were deemed eligible?
44. From how many records was data extracted
45. Measurement of reviewer inter-reliability in data extraction
46. Kappa reported for data extraction
47. Screening software used?
    1. Y/N
    2. Produce, if named: _______________
48. Was Crowd screening or machine learning mentioned?
    1. Y/N, If yes:
       1. Crowd
       2. Machine
       3. Both
49. Full text retrieval (click all that apply)
    1. Human
    2. Software aided
50. Reported providing training to investigative team members performing review – Y/N
    1. Test set
    2. Training manual
    3. One-on-one
    4. Other
    5. Not clearly reported
51. Were reviewers evaluated
    1. When
       1. Outset
       2. End
    2. Where
       1. Abstract – Y/N / unclear
       2. Full text – Y/N / unclear
       3. Data extraction -Y/ N / unclear
52. Acceptable threshold reported – Y/N
    1. Text box
53. Reported approach to conflict resolution – Y/N
    1. Resolved by PI
    2. Resolved through discussion
    3. Resolved by a third/additional reviewer
    4. Resolved by combination of discussion and third/additional reviewer
    5. Other
54. Funding for completing the SR
    1. Non-profit
    2. For profit
    3. Mixed
    4. Author reported Unfunded
    5. Not reported
55. ICMJE authorship
    1. Statement on whether all authors read and approved the manuscript?
       1. Draft the work
       2. Critically appraising if for important intellectual content
       3. Finally approval of the version
       4. Agreement to be accountable for all aspects of the work in ensuring that questions related to the accuracy or integrity of any part of the work are appropriately investigated and resolved.
56. Types of analysis used
    1. Descriptive or narrative analysis
    2. Meta-analysis
    3. Meta-regression
    4. Other aggregation
    5. Statistical heterogeneity
    6. Risk bias
    7. Publication bias

Results

1. Number of citations screened – title/abstract
2. Number of citations screened – full text
3. Number of eligible citations – data extracted
4. Report of conflict rate – Y/N
   1. Text
5. Report of kappa – Y/N
   1. Method used
   2. Results
6. Estimate of time to study completion, using
   1. Registered protocol
   2. End point of search to manuscript submission
   3. Search update performed
      1. Once
      2. Twice

**Supplementary Material 3: Number of systematic reviews included in each section of the reported results**

| Analyses |  | N (%) |
| --- | --- | --- |
| Relationship between screener number and initial citation set size | SRs out of sample of 259 reporting the total number of screeners contributing to the initial level of screening. | 192 (74.1%) |
| Average workload per screener at title/abstract | SRs out of sample of 259 reporting using two assessments per citation during title/abstract screening and provided a citation set size at the title/abstract level. | 156 (60.2%) |
| Average workload per screener at full text | SRs out of sample of 259 reporting workload per screener at full text | 139 (53.7%) |
| Methodological Approach to Title screening | SRs out of sample of 259 who reported performing title only screening before proceeding to abstract screening | 42 (16.2%) |
| Methodological Approach to Title and Title/Abstract screening | SRs out of sample of 259 reporting screening methods used for title and title/abstract screening | 214 (82.6%) |
| Methodological Approach to Full-text screening | SRs out of sample of 259 reporting screening methods used for full-text screening | 186 (71.8%) |
| Methodological Approach to Data Abstraction | SRs out of sample of 259 reporting methods for data abstraction | 259 (100.0%) |

**Supplemental Material 4. Relationship between initial citation set size (log scale) and using more than 2 screeners for the initial screening level.** Dots at 1 (or 0) represent systematic reviews which used (or did not use) more than 2 screeners. The blue curve is a binomial 5-knot restricted cubic spline with a 95% shaded confidence band. The vertical line indicates a citation size of 2500.


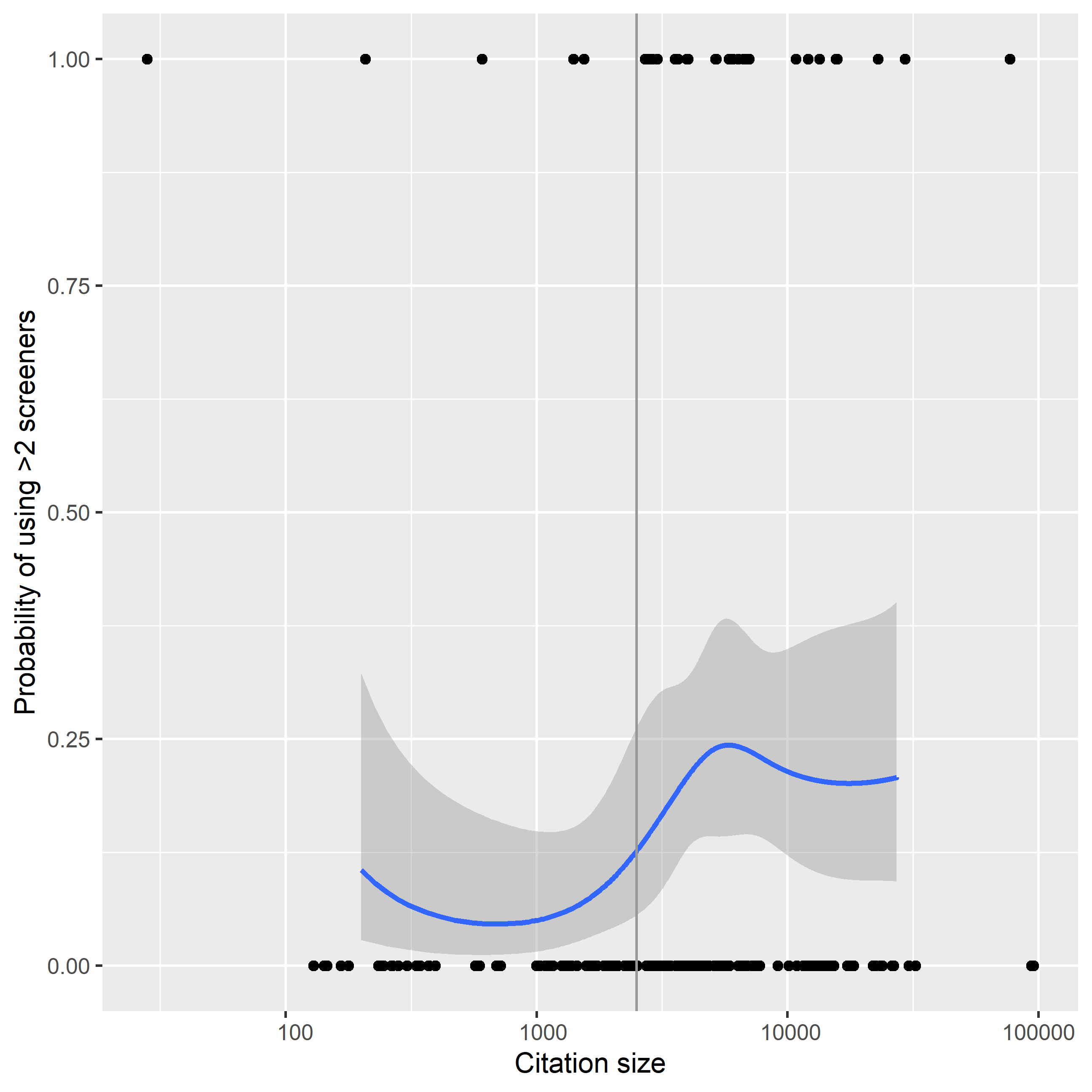


**Supplementary Material 5. Relationship between initial citation size (log scale) and using the gold-standard approach for citation screening at the title/abstract level.** Dots at 1 (or 0) represent systematic reviews which used (or did not use) the gold-standard approach. The blue curve is a binomial 5-knot restricted cubic spline with a 95% shaded confidence band. The vertical line indicates a citation size of 2500.


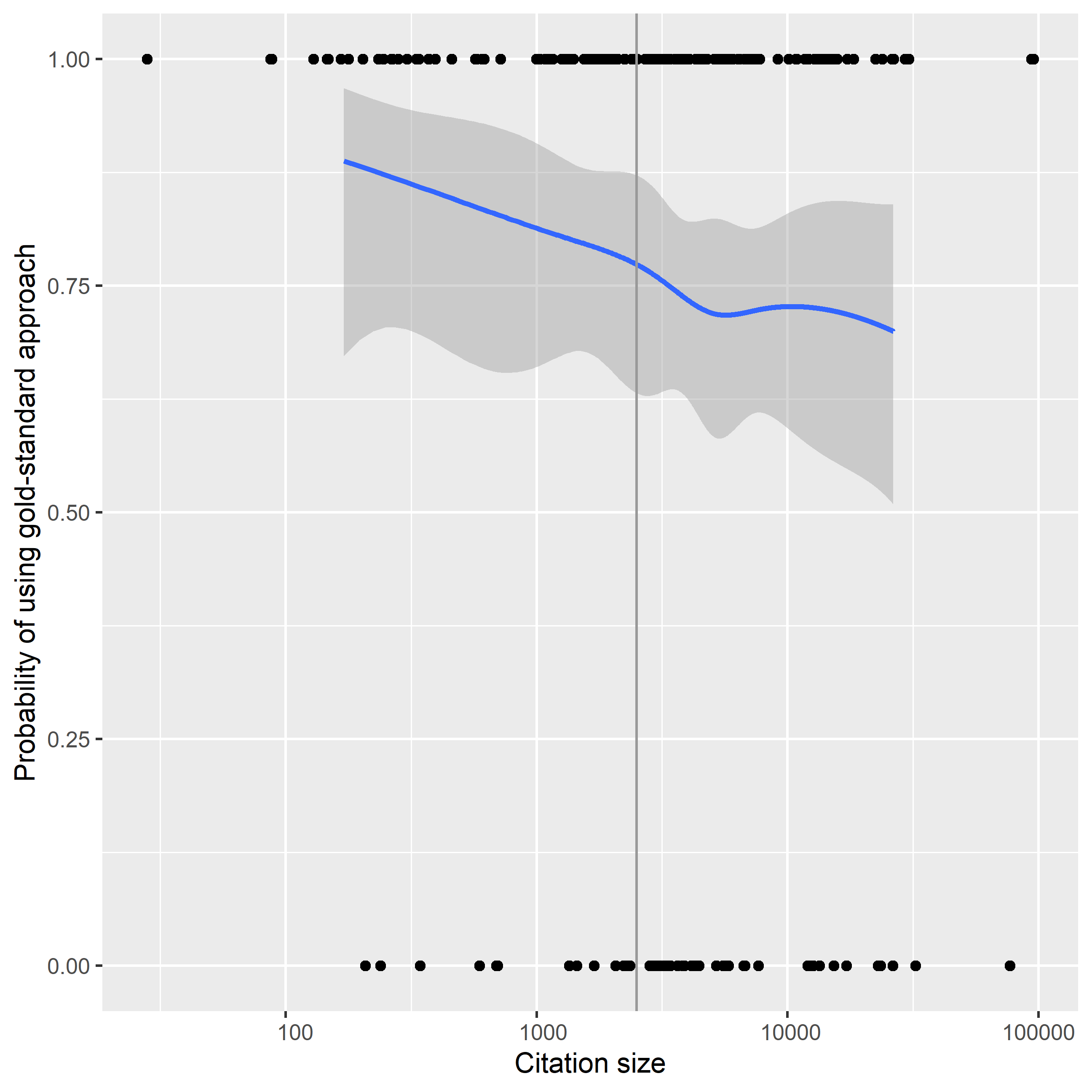


**Supplementary Material 6. Distribution of Methodology Used during Full text Screening by Terciles Created Using Full Text Citation Size (n = 186)^a^**

|  | **N** | **Gold-standard methodology**  **N = 147**  **N (%)** | **Two assessments/citation, not independent or not stated**  **N = 22**  **N (%)** | **Single assessment/citation**  **N = 17**  **N (%)** |
| --- | --- | --- | --- | --- |
| Low (2 to 53 citations) | 62 | 57 (91.9) | 4 (6.5) | 1 (1.6) |
| Mid  (54 to 150 citations) | 62 | 42 (67.7) | 11 (17.7) | 9 (14.5) |
| High  (154 to 1873 citations) | 62 | 48 (77.4) | 7 (11.3) | 7 (11.2) |

^a^This table is limited to the citations that reported their screening approach thus the total number of SRs included is 186

**Supplementary Material 7. Relationship between full-text citation size (log scale) and using the gold-standard approach to screening at the full text level.** Dots at 1 (or 0) represent systematic reviews which used (or did not use) the gold-standard approach. The blue curve is a binomial 5-knot restricted cubic spline with a 95% shaded confidence band.


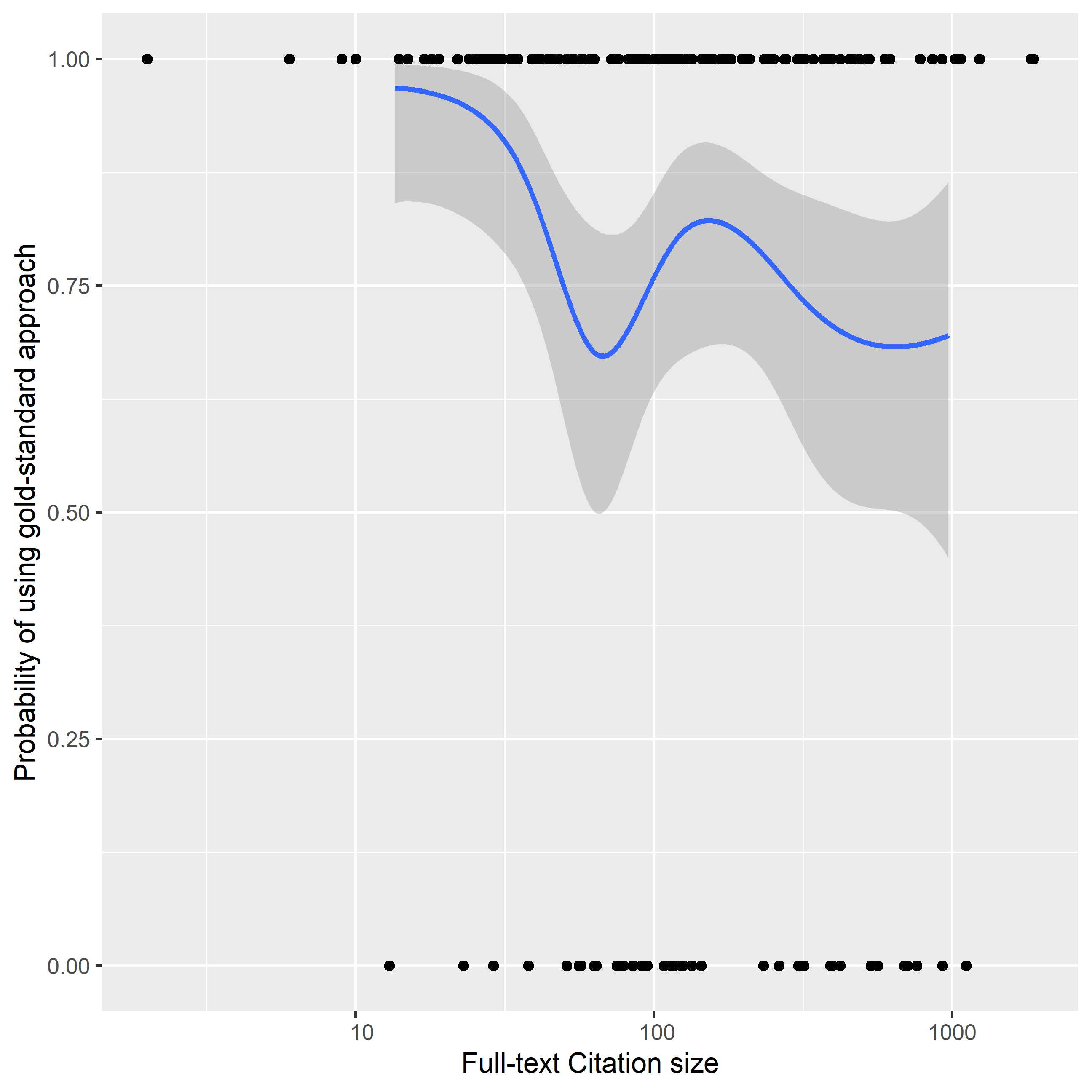


**Supplementary Material 8. Methodology Used during Data Extraction by Initial Citation Set Size**

| **Initial citation size category** |  | **Methodology Used during Data Extraction** | | | | |
| --- | --- | --- | --- | --- | --- | --- |
|  | **N** | **Majority/all data extracted in duplicate**  **N = 141**  **N (5)** | **Single extraction with verification from a second review**  **N = 34**  **N (%)** | **Single extraction**  **N = 16**  **N (%)** | **Other^a^**  **N = 2**  **N (%)** | **Not reported**  **N = 66**  **N (%)** |
| <1000 | 50 | 27 (54.0) | 3 (6.0) | 2 (4.0) | 0 (0.0) | 18 (36.0) |
| 1000-2500 | 50 | 29 (58.0) | 7 (14.0) | 3 (6.0) | 1 (2.0) | 10 (20.0) |
| 2500-5000 | 58 | 34 (58.6) | 5 (8.6) | 3 (5.2) | 0 (0.0) | 16 (27.6) |
| 5000-10000 | 50 | 27 (54.0) | 11 (22.0) | 4 (8.0) | 1 (2.0) | 7 (14.0) |
| >10000 | 51 | 24 (47.1) | 8 (15.7) | 4 (7.8) | 0 (0.0) | 15 (29.4) |

^a^Other includes: No eligible studies identified, so data extraction not performed (n = 1); combination of duplicate and single extraction depending on data type (n = 1)

**Supplementary Material 9. Methodology Used during Data Extraction by Terciles Created using Data Extraction Citation Set Size**

| **Data extraction citation size category** | **N** | **Methodology Used during Data Extraction** | | | | |
| --- | --- | --- | --- | --- | --- | --- |
|  |  | **Majority/all data extracted in duplicate**  **N = 141**  **N (%)** | **Single extraction with verification from a second review**  **N = 34**  **N (%)** | **Single extraction**  **N = 16**  **N (%)** | **Other^a^**  **N = 2**  **N (%)** | **Not reported**  **N = 66**  **N (%)** |
| Low  (1-31 citations) | 84 | 45 (53.6) | 10 (11.9) | 4 (4.8) | 1 (1.2) | 24 (28.6) |
| Mid  (32-58 citations) | 88 | 43 (48.9) | 13 (14.8) | 7 (8.0) | 1 (1.1) | 24 (27.3) |
| High  (59-103 citations) | 87 | 53 (60.9) | 11 (12.6) | 5 (5.7) | 0 (0.0) | 18 (20.7) |

^a^Other includes: No eligible studies identified, so data extraction not performed (n = 1); combination of duplicate and single extraction depending on data type (n = 1)
